# Building the Partners HealthCare Biobank at Partners Personalized Medicine: Informed Consent, Return of Research Results, Recruitment Lessons and Operational Considerations

**DOI:** 10.3390/jpm6010002

**Published:** 2016-01-14

**Authors:** Elizabeth W. Karlson, Natalie T. Boutin, Alison G. Hoffnagle, Nicole L. Allen

**Affiliations:** 1 Brigham and Women’s Hospital, Boston, MA 02115, USA; nmallen@partners.org; 2 Partners HealthCare System, Boston, MA 02115, USA; nboutin@partners.org; 3 Massachusetts General Hospital, Boston, MA 02114, USA; ahoffnagle@partners.org

**Keywords:** Biobank, Partners Biobank, biorepository, personalized medicine, precision medicine, Partners HealthCare Biobank, electronic informed consent

## Abstract

The Partners HealthCare Biobank is a Partners HealthCare enterprise-wide initiative whose goal is to provide a foundation for the next generation of translational research studies of genotype, environment, gene-environment interaction, biomarker and family history associations with disease phenotypes. The Biobank has leveraged in-person and electronic recruitment methods to enroll >30,000 subjects as of October 2015 at two academic medical centers in Partners HealthCare since launching in 2010. Through a close collaboration with the Partners Human Research Committee, the Biobank has developed a comprehensive informed consent process that addresses key patient concerns, including privacy and the return of research results. Lessons learned include the need for careful consideration of ethical issues, attention to the educational content of electronic media, the importance of patient authentication in electronic informed consent, the need for highly secure IT infrastructure and management of communications and the importance of flexible recruitment modalities and processes dependent on the clinical setting for recruitment.

## 1. Introduction

The first decade of the 21st century was remarkable for the explosive growth in our knowledge of the human genome through the advances in genotyping technology and the discoveries of genetic associations through genome wide-association studies. The second decade has been marked by rapid advances in translating genetic discoveries into targeted therapeutics based on genotype-phenotype associations and biologic mechanism research, particularly in the field of cancer treatment, known as “precision medicine”. In 2010, Partners HealthCare launched an ambitious program, the Partners HealthCare Biobank (Partners Biobank), to collect DNA, plasma and serum samples from 75,000 patients with diverse phenotypes and consent from patients for the linkage between samples and detailed clinical data in the electronic medical record (EMR), together with lifestyle, behavior and environmental risk factors, along with family history data collected by survey. The goals were to provide a foundation for the next generation of translational research studies of genotype, environment, gene-environment interaction, biomarker and family history associations with disease phenotypes. In this chapter, we review the close integration with our institutional review board on designing informed consent, electronic informed consent and patient education materials and addressing issues of privacy and the return of research results. We provide information on the design and success of an electronic consent process and the lessons learned from in-person and electronic recruitment implementation.

## 2. Partners HealthCare Biobank Informed Consent

The goal of the Partners Biobank is to obtain consent from patients for the collection of blood samples (for serum, plasma and DNA/genomic research), the linkage of samples to extensive clinical data in the electronic medical record and data on lifestyle, behavioral and environmental factors, as well as data on family history collected by survey. Subjects consent to: (1) provide 30 to 50 cc of blood collected as part of clinical care (adding 3 to 5 tubes to a clinical draw), or collected as a research draw; (2) allow researchers at Partners HealthCare to use samples for any type of research on human health, including genomics, biomarker analyses, epidemiology and outcomes research and the creation of cell lines; (3) use coded samples linked with de-identified variables (e.g., age, but not date of birth) or use coded samples linked to identified data from the EMR, including electronic clinical notes with appropriate IRB approval; (4) re-contact by IRB-approved Partners investigators for additional data or samples; (5) the return of actionable genetic research results; and (6) share genomic results with national databases, such as dbGAP. The Partners Biobank has obtained a Certificate of Confidentiality. Subjects are offered the options of providing consent, refusing consent or remaining undecided via traditional in-person recruitment by trained research assistants or via electronic informed consent (eIC) via email through a patient portal linked to the eIC website. 

## 3. Impact of Working with Institutional Review Board Leaders on Informed Consent Issues in Biobanking 

The Partners Biobank has worked closely with the Partners Human Research Committee (PHRC) (the Institutional Review Board) to ensure that its procedures meet ethical standards for human subject research. As with all human research projects, the Biobank must submit all protocols and external-facing communications to the PHRC for review and approval. In light of its scale and the novelty of some of its operations, the Biobank has been actively engaged since 2009, prior to launching the project in 2010, in an interactive dialogue with PHRC leadership to review recruitment and operational plans before they are formally documented and submitted for review. The PHRC leadership and Biobank staff meet every couple of months to review planned changes to the Biobank protocol and to discuss and design novel procedures. For example, the concepts of return of research (RoR) results pertaining to genomic results and of electronic informed consent (eIC) were thoroughly discussed and reviewed with the PHRC before formal plans were set in motion. In the case of eIC, a paper mock-up of the planned eIC tool was created in close collaboration with the PHRC. For several months, monthly meetings enabled iteration on the website content and the definition of detailed functional requirements. Some of the questions discussed during these meetings addressed the best ways to translate in-person informed consent processes into a multimedia experience. Some of the issues that were discussed at length include:

- How many times should the Biobank send email invitations to patients?
- Does eIC require that consenting subjects authenticate their identity only at initial log-in or authenticate a second time after electronically agreeing to consent?
- Should patients who consent electronically be provided a paper-copy of the consent form, as typically, patients must retain a copy, or is it adequate to email a PDF of the signed consent form?
- Should patients be quizzed on the consent form content to ensure that they understand it?
- Should patients have to click a button after each section in the consent form to demonstrate that they are reading each section?

The close collaboration between Biobank staff and PHRC leadership has ensured that the Biobank can rapidly move forward with the tools and procedures required to scale to 75,000 subjects while ensuring the integrity of the informed consent process.

## 4. Developing a Return of Research Results Policy

Since 2009, when the Partners Biobank was in the planning stage, the PHRC and Biobank leaders have engaged in discussions of issues around RoR informed in part by the changing landscape nationally [1,2].

During a pilot recruitment phase, the Biobank conducted focus groups and surveys around the issue of RoR. We found that 89% of subjects preferred to receive results if a genetic finding was related to a serious condition and there was an action that could be taken to prevent disease, a so-called “actionable” result [3]. As per published guidelines [4], we developed a policy and procedure around genetic research findings. The PHRC protocol specifies that if an investigator finds a potentially actionable result, he/she is to notify the PHRC and the Biobank Executive Committee, who will review the latest evidence regarding penetrance, disease associations and clinical implications for disease prevention of drug treatment choice and designate the finding as actionable, potentially actionable or not currently actionable. The RoR process will be triggered only when clinically-important results are defined as results with “important health implications for the participant, and when the associated risks are established and substantial.” The Biobank will only return research results with clear evidence of pathogenicity, substantial penetrance and an actionable next step (e.g., established value for surveillance, cascade screening, preventive measures and/or treatment). The Executive Committee will notify the PHRC about the research finding. Since the blood samples are not collected via a certified clinical laboratory (CLIA) approved process, the Biobank will inform the subject with a general letter stating that a potentially meaningful research result has been found and that an appropriate practitioner (Doctor of Medicine (MD), Nurse (RN), or Clinical Genetic Counselor (CGC)) who understands the health-related research findings will contact them to discuss their research result. Subjects may decide not to get this information (opt-out) when they talk to the healthcare professional. During this discussion, subjects will be told that the research findings will need to be repeated using a CLIA to validate the findings using an independently-drawn sample from them. The practitioner will offer to provide the result to the subject’s healthcare provider, who will be provided names of clinical laboratories that can validate the research results in an independent sample from the subject. If clinically indicated, the provider will draw a second blood sample as a CLIA sample to confirm the finding. If clinically determined, it is possible that the patient’s health insurance company will reimburse the test if the patient meets the company’s criteria for reimbursement. Research results do not go into the EMR. However, results from the CLIA testing may be placed into the EMR.

## 5. Recruitment Successes and Failures: What Have We Learned? 

The Partners Biobank recruitment activities take place within the founding hospitals of Partners HealthCare: Brigham and Women’s Hospital (BWH) and Massachusetts General Hospital (MGH), located in the greater Boston area. The benefits of recruiting in these two academic medical centers are that improving care through research discovery is part of their mission, they are regionally proximate, which allows for central banking and storage, and they have various types of primary care, specialty care, inpatient and outpatient units, which allow for diverse patient populations to be approached. Recruitment for the Partners Biobank launched in 2010, and over 30,000 patients have been enrolled as of October 2015. The goal is for 75,000 subjects to be enrolled by 2018.

Recruitment for a large-scale biobank can be conducted in a variety of ways. The Partners Biobank has leveraged many types of approaches. Using teams of dedicated research assistants at each hospital site, the Partners Biobank has been able to effectively enroll subjects in-person in the context of outpatient visits, inpatient stays and emergency department settings. Some biobanks have leveraged mail contact to efficiently recruit patients [5,6,7]. Others have utilized mailings prior to clinical visits augmented with personal contact by research assistants. The Partners Biobank, in collaboration with the PHRC, determined that an optional mail contact supplemented by an in-person meeting with a dedicated research assistant at the patient’s visit is an appropriate method for recruitment. Some eligible patients receive letters in advance of upcoming appointments describing the study. In high volume clinics, patients are directly approached in the waiting room without receiving a letter (so-called “same-day consent”). Letters are co-signed by the study Principal Investigator and the patient’s healthcare provider. The benefits to contacting patients about the Biobank by mail are that patients have an opportunity to learn about the study before they encounter the research assistant in the clinic and the study is being described to them in a letter co-signed by their own provider. The challenges to mail contact are the work required to get the mailings sent out regularly prior to patients’ visits, the potential that many patients do not read the letters and that at our institutions, up to 40% of patients reschedule visits after the mailing has been sent out, thus negating the timing of the mailing. 

At the visit date, research assistants work closely with clinic staff to identify the most appropriate time to speak to eligible patients about the project and to obtain informed consent without interfering with clinic flow. Some clinics feature signs and brochures advertising the Biobank, while others clearly separate research efforts from the clinical enterprise. In many cases, research assistants have the initial conversation in a public waiting room where patients have anywhere between five minutes to a few hours to wait before being taken back to an exam room. In the initial recruitment spiel, research assistants describe the purpose of the project, what participation involves and how the project aims to help further medical discovery and personalized medicine. This two to five minute spiel is designed to spark patient’s interest in the Biobank, so a further discussion about the informed consent document may ensue. A consent form fact sheet that provides definitions and details to answer frequently-asked questions is provided with the informed consent document (Table 1). After patients are allowed ample opportunity to review the consent form and fact sheet and after their questions are answered, research assistants may obtain consent in the waiting room, a private or semi-private space, pending the patient’s preference. At this time, patients may choose to provide consent, decline consent or take material home to review later. If at any point in the recruitment process the patient is called to resume their clinical visit, the research assistant stops and resumes the conversation after the patient’s visit to seamlessly integrate with the clinical flow.

**Table 1:** Partners Biobank informed consent form and fact sheet content.

Consent Section	Consent Form Topic	Fact Sheet Topic
Purpose	Study how genes and other factors contribute to disease	Yes
Procedures	Fresh blood sample (up to 5 tubes) and future discarded specimens (blood, urine, tissue)	Yes
	Samples linked to electronic health record	Yes
	Questionnaires about health behaviors and family history	Yes
	Re-contact for other information or studies	No
Research Conducted	Biological and genetic research	Yes
	Cell lines and pluripotent stem cells may be created	Yes
Return of Results	Unlikely, but may receive research result of high medical importance	Yes
	Patient and insurer may be responsible for costs of tests and follow-up care	No
Benefits	No direct benefit, but may help us understand, prevent, treat or cure disease	Yes
	No payment for samples	Yes
Sample and Information Storage	Samples are de-identified, and key to code is stored securely	Yes
Researchers with Access	Partners investigators	No
	Researchers at non-Partners institutions	No
	For-profit companies that work with Partners investigators	No
	Central banks who may share coded samples and data with other researchers	No
	Samples will not be sold for profit	No
Withdrawing	Can withdraw anytime, but it is not possible to destroy samples and information that have already been given to researchers	No
Risks	Potential loss of privacy	Yes
	Influence on insurance companies and/or employers	No
	Cannot predict how genetic information will be used in the future	Yes
	Bruising or infection from blood draw	No
Certificate of Confidentiality	Researchers cannot be forced to disclose identifying information, even by a court subpoena	Yes
	Does not prevent patient from voluntarily releasing information about self-involvement in research	Yes
	Certificate does not prevent researchers from disclosing information without consent in incidents, such as child abuse and intent to harm self or others	No

Inpatient recruitment imposes fewer demands on research assistants’ timing around clinical interventions, but offers a smaller patient volume than many outpatient settings provide. In an inpatient setting, research assistants collaborate with the charge nurse or individual patient’s nurses to determine who is appropriate for approach (patients who are not heavily sedated and who are able to provide informed consent) and approach patients in their room between clinical interventions, after a meal and when the patient is not sleeping. The nature of the inpatient setting allows for more flexibility for when research assistants may approach eligible patients, but low turnover limits the consent numbers of the overall consenting patients from those sites. If an eligible patient is not available at the first time of approach, a research assistant may return to the floor at a later date or time, thus ensuring that there is no disruption with the patient’s clinical care. In some cases, however, identifying appropriate times to approach inpatients can be challenging, particularly in inpatient units that require more frequent clinical intervention, such as on the orthopedic inpatient floors.

As demonstrated by other biobanks, primary care sites are some of the most effective recruitment locations for the Partners Biobank due to the high patient volume and patient willingness to join [6]. Recruiting in primary care clinics also has other advantages. Primary care has a high percentage of patients for whom clinical phlebotomy is ordered (and research tubes can be added to a clinical draw), thus reducing the barrier for patients to undergo specific phlebotomy for research samples. The diversity in the patient population of primary care clinics helps reduce sampling bias, which has been found to be a problem for some biobanks [8]. Other productive recruitment sites are high volume clinics, including surgical practices, such as orthopedics and locations where physicians fully support the mission of the Biobank and speak to their patients about the importance of joining. The Biobank has also set up central sites within the hospitals where patients can come and learn more about the project, provide consent, provide the blood sample or find out how to engage with the project. The Biobank has hosted monthly “DNA Days” to engage volunteers from staff and patients, typically in a lobby location at each hospital.

The Partners Biobank constantly seeks support from different departments and divisions at each of the participating hospitals in order to broaden the number of eligible patients that research assistants can approach. Launching a new clinic site requires identifying a champion physician who supports the Biobank’s mission, working out the logistics of having research activities in that clinical setting and taking into account unique characteristics of the patient population that may require tailored recruitment strategies. For example, in order to launch primary care as a recruitment site at BWH, the primary care providers required that the Biobank develop additional educational information written at a middle school level to be specifically appropriate for their patient population. When it comes to clinic expansion, the Biobank takes into consideration many factors, including the clinic’s level of support for research activities, patient volume, number of providers, percentage of patients who get clinical phlebotomy, patient interest in research, patient population and space for research assistants to conduct a private or semi-private discussion if needed. Each of these characteristics is used to help the Biobank determine whether engagement with that clinic site will lead to high numbers of consenting or collected subjects or the potential to engage with a new patient population that has not been previously represented in the Biobank.

There are unique challenges to enrolling vulnerable populations into biobanks, including the need for the presence of an appropriate surrogate to provide surrogate consent and the potential need to re-consent pediatric patients at the age of maturity [9,10]. The Partners Biobank is considered a “minimal risk” study. To maintain this level of risk, the Biobank does not directly enroll vulnerable populations, including pediatric patients and patients who cannot provide consent on their own behalf. 

Engaging a diverse patient population is required to enable generalizable and unbiased research [8]. There is research, however, that demonstrates that patients of certain demographic backgrounds may be less likely to participate in banking projects, thus creating an inherent sample bias [8]. This difference may not just be reflective of certain patients’ willingness to join research, but may be a feature of biobanks specifically. When compared to a National Survey Database, the Mayo Clinic Biobank noted a difference in the demographic background of subjects who provided consent, even after restricting the national database to the catchment region for the Biobank [6]. There is also evidence, however, that members of racial and ethnic minority groups who do join biobanks generally join out of an altruistic desire to help future generations, the same reason as stated among primarily white populations [11,12]. The Biobank strives to enroll patients of diverse racial and ethnic backgrounds by ensuring that all marketing and recruitment material features pictures of people from diverse backgrounds, recruiting in sites where there is a higher prevalence of patients from underserved populations and by holding volunteer events targeted at the employee population, which is racially and ethnically diverse.

A critical tenant of the Biobank is to enroll a patient population that is reflective of the patients who seek care at Partners HealthCare institutions. To promote patient inclusion regardless of patients’ primary language, all recruitment and consent materials are fully translated into Spanish, and the Biobank employs research assistants who can speak to patients in English or Spanish. The Biobank leverages hospital interpreters to obtain consent across 11 other language groups for which an IRB-approved short consent form is available. To encourage racial and ethnic diversity, the Biobank launched recruitment in primary care, the Spanish clinic and in a mostly Spanish language speaking community health center. Regarding the overall demographics of the Biobank, they reflect the distribution of the race/ethnicity of the population who received care at these hospitals (Table 2).

Obtaining informed consent is critical for the Partners Biobank as an opt-in, fully-consented initiative. In collaboration with the PHRC, the Biobank developed a five-page consent document and an associated fact sheet that includes all required elements of informed consent for a biobanking project (Table 1) [13]. The fact sheet is an educational document that includes expanded information about the informed consent document, as well as relevant definitions. The fact sheet is part of the PHRC approved consent process and is designed to help patients comprehend key elements of consent. Patients have the option of reading a paper version of the consent form and associated fact sheet on paper or on an iPad (so-called “paperless consent”) with a research assistant present to answer questions and concerns. Primary patient concerns include privacy, privacy of their genetic information, return of research results and how to get blood drawn. Research assistants are trained to respond to patient inquiries about all of the topics covered in the consent form to help ensure patients are able to make an informed decision about joining the project. Primary reasons for refusal include that patients do not want a research blood draw, have privacy concerns or have no stated reason (Table 3). 

**Table 2:** Demographics of the Partners Biobank.

Total N	30,066
Age	
Mean age	57.6
Gender	
Female	58%
Male	42%
Race/ethnicity	
White	83%
Black	6%
Hispanic	4%
Asian	2%
Other/Unknown	5%

**Table 3:** Reasons for refusal to the Partners Biobank.

Reasons for Refusal	N	%
Unknown	2455	22.9
Reason not given	2386	22.3
Does not want a blood draw	1247	11.6
Does not like research	832	7.8
Privacy Concerns	798	7.4
Other	786	7.3
Busy	557	5.2
Patient is sick	386	3.6
Not interested	364	3.4
Privacy concerns regarding genetic data	239	2.2
In too many studies	178	1.7
Refuses to be re-contacted	178	1.7
Other	314	3.0

The Partners Biobank has effectively integrated research recruitment into the clinical workflow at two academic medical centers. Patients have responded positively to the initiative, and those patients who choose to refuse consent do so for a variety of reasons. Research assistants make every effort to ensure that patients from all backgrounds are able to make informed decisions about joining the initiative. With continued support from key stakeholders, including Partners HealthCare and hospital leadership, clinicians, researchers, staff and patients, the Partners Biobank expects to meet recruitment goals.

## 6. Designing Electronic Educational Tools and Electronic Consent Methods

Electronic informed consent (eIC) was conceived of as a strategy for meeting recruitment targets while managing Biobank costs, all while maintaining the integrity of the informed consent process. Multimedia experiences have complemented classroom teaching for decades; online learning or e-learning has become a multi-billion dollar business [14], and financial transactions and purchases online are common and expected experiences. The informed consent process appeared to be a natural candidate for translation into a multimedia environment. Moreover, it appeared that eIC might have the potential to actually enhance the informed consent experience for participants. The use of interactive and multimedia can help promote an individual’s understanding and confidence in their understanding of Biobank consent [15]. eIC can also provide patients with more time for decision making [16].

**Figure 1:**
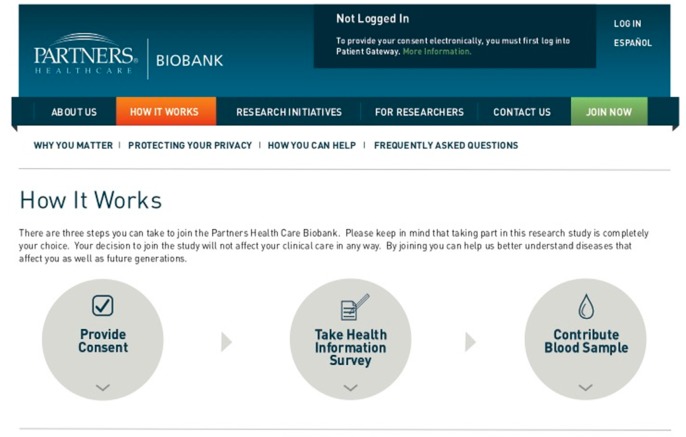
Website image (“How It Works” page).

At Partners HealthCare, eIC is performed on a website [17] that describes how the Partners Biobank works and provides educational material and resources for patients to review (Figure 1). The website features a multimedia version of the Biobank consent form. While the paper version of the consent form is five pages long, the electronic version displays the same content on three pages. The baseline content is enhanced with contextual information. Key words in the consent form are underlined, and clicking an underlined word displays its definition. Moreover, additional context is provided in conjunction with each section in the consent form. Potential subjects must review each page in the consent form and then indicate that they are providing consent by clicking a radio button. This triggers the display of a confirmation pop-up to validate that the subject understands that consent is being provided. Subjects are then driven to a confirmation page from which the health information survey may be filled out, and instructions on how to provide a blood draw are detailed. 

The eIC website features several sections that are designed to enhance a patient’s understanding of the Biobank. A “frequently asked questions” page addresses questions that patients typically ask of Biobank staff during the in-person consent process. A section on research initiatives provides lists and describes some of the studies that are leveraging the Biobank for their research. The design of the website is clean and simple. The branding style was derived from the Partners HealthCare style guide.

A few functional and technical requirements are essential to the success of eIC. They are identity authentication, security, user experience and marketing. Authentication refers to the process that enables the Biobank to ascertain the identity of the subject who provides consent. There are several ways to enable the unique identification of subjects. For example, it is possible to purchase the services of an identity verification service. Such services may verify identify information against authoritative sources, such as credit bureaus or other databases. At Partners HealthCare, we decided to leverage the unique user name and password that are required when patients set up accounts with the patient online portal (Patient Gateway) at Partners HealthCare. The Biobank’s ability to validate a subject’s identity with a unique user name and password was essential to the development and success of eIC.

A second requirement is security. The eIC tool must maintain a subject’s confidentiality by ensuring that standard security and encryption procedures are followed. eIC at Partners HealthCare makes use of standard web security measures, such as secure network connections between client (browser) and server and between the application and enterprise services. Web servers are kept up to date with security and OS-level patches. Data are stored in an encrypted state. Moreover, the application is periodically subjected to a security assessment by an external application security vendor.

The Biobank made a decision early in the process to hire user experience experts to design the eIC tool. While eIC might be as simple as posting a consent form in Portable Document Format (PDF) with a series of radio buttons to indicate consent, we wanted to ensure that the subject would become immersed in an educational multimedia experience. As a result, we produced a couple of short videos: (1) an overview of the Partners Biobank; and (2) an overview of privacy procedures. Moreover, an interactive agency designed the user interface and graphics for the eIC website. 

The fourth requirement to the success of eIC is the ability to draw large numbers of people to the eIC tool. At Partners HealthCare, an email campaign enables the Partners Biobank to send email to every patient with a Patient Gateway account and an upcoming clinical appointment. It could be possible to leverage other marketing strategies to drive patients to eIC, such as targeted ads, assuming such strategies were approved by the PHRC. The operation behind the Partners Biobank’s email campaign required the implementation of an application to generate and manage the lists of patients to whom emails are sent. This application generates patient lists based on upcoming appointment, then filters patients out based on existing Partners Biobank consent statuses, age, vital status and whether the patient has already received a Partners Biobank email. This tool manages the frequency of emails, as well as the number of emails that a patient may receive. The Partners Biobank is currently IRB approved to send three emails in one year and a fourth follow-up email in the following year. Patients provide consent after each of the three emails we have been sending to date. Questions and complaints are managed by a help desk application that enables email contact to Partners Biobank staff who can respond within business hours. Staff track the content of messages to enable future improvements in Partners Biobank processes. 

From June 2014 through August 2015, eIC at Partners HealthCare enabled the enrollment of 5594 subjects into the Partners Biobank. The rate of consent is 3.5% of all emailed patients and 29% of logged-in patients. The requirement to log-in to the patient portal to view the consent form appears to be a major barrier to consent. Once the patient provides their log-in credentials, the consent rate improves dramatically from 3.5% to 29%. The Partners Biobank sends first emails to 1000 to 8000 patients each week. While we consider our electronic consent strategy to be a success, we cannot qualify this success because there are no benchmarks, yet, to compare our experience. This should be changing soon as more healthcare systems launch their own eIC tools and processes, especially since President Obama’s Precision Medicine Initiative (PMI) working group recommends electronic consent as a recruitment method.

The primary benefits of eIC have been an increased enrollment into the Partners Biobank and a vast increase in the scale of the Partners Biobank’s outreach effort. To date, the Biobank has sent email to nearly 160,000 patients. From June 2014 through August 2015, eIC accounted for 34% of enrolled subjects and 19% of all Biobank participants. This outreach effort has resulted in greater awareness of the Partners Biobank by patients who provide their consent in-person in a clinic. After a one-time investment, eIC has also enabled the Partners Biobank to enroll thousands of subjects without significant operational cost. 

While eIC is a feasible and potentially game-changing strategy for research studies that depend on patient recruitment, it does present some challenges and limitations. A key limitation is the dependence on a robust infrastructure to authenticate each subject’s identity. At Partners HealthCare, this has limited the pool of potential participants to those with Patient Gateway accounts, which is only a fraction of the full patient population. A second limitation is that drawing large numbers of patients to the eIC website requires sophisticated tools and policies to manage electronic messaging with patients. A third limitation is that the patient population that enrolls via eIC is generally less diverse than the population that enrolls via in-person consent (Table 4). Due to the successes and limitations of eIC, the Partners Biobank sees a continued role for both in-person and electronic consent recruitment strategies to maximize Partners Biobank enrollment.

**Table 4:** Comparison of in-person informed consent and electronic informed consent demographics for the Partners Biobank.

	In-Person	eIC	Total	*p*-Value
Number	24,472	5594	30,066	
Age				0.2216
Mean age	57.6	57.5	57.6	
Gender				0.0005
Female	58%	61%	58%	
Male	42%	39%	42%	
Race				<0.0001
Asian	2%	2%	2%	
Black	7%	1%	6%	
White	81%	92%	83%	
Hispanic	5%	1%	4%	
Other/unknown	5%	3%	5%	
Education				< 0.0001
8th grade or less	1%	0%	1%	
Some high school	2%	0%	2%	
High school/GED	18%	6%	16%	
Some college	6%	3%	5%	
Graduated College	52%	73%	56%	
Graduate school	2%	3%	2%	
Unknown	19%	15%	18%	

## 7. Conclusions

The Partners HealthCare Biobank has obtained consent from >30,000 subjects for research studies performed by Partners investigators and collaborators to provide a large biobank of serum, plasma and DNA samples linked to extensive clinical data and survey data. A research Biobank Portal database contains coded data on consenting biobank subjects, including demographic data, diagnoses (e.g., ICD-9/ICD-10 codes), procedures (e.g., CPT codes), pharmacy data, inpatient and outpatient encounter information, provider information, laboratory data, available sample types and genotypes and subject survey data on health behaviors, such as smoking, as well as phenotypes defined by bioinformatics algorithms and healthy controls derived using a validated index. Researchers may query the database using the Biobank Portal online query tool for any of these data types. They may also design and download limited datasets for subjects of interest and may make requests for samples and genomic data using the Portal. If necessary, detailed medical record information can be obtained with proper IRB approval. The Partners Biobank has, as of October 2015, fulfilled 77 investigator requests for Partners Biobank samples and/or data. More than 140 investigators have accessed the Biobank Portal since its launch in May 2015. By working closely with PHRC leaders, issues around broad consent, privacy, data sharing and return of research results were discussed and agreed upon with Partners Biobank leaders. Lessons learned include the need for careful consideration of ethical issues, attention to the educational content of electronic media, the importance of patient authentication in eIC, the need for highly secure IT infrastructure and management of communications and the importance of flexible recruitment modalities and processes dependent on the clinical setting for recruitment. The Partners Biobank has developed a novel approach to obtaining electronic informed consent that can provide a model for other biobanks in the future.
